# Social Work Staffing and Use of Palliative Care Among Recently Hospitalized Veterans

**DOI:** 10.1001/jamanetworkopen.2022.49731

**Published:** 2023-01-04

**Authors:** Portia Y. Cornell, Christopher W. Halladay, Anna-Rae Montano, Caitlin Celardo, Gina Chmelka, Jennifer W. Silva, James L. Rudolph

**Affiliations:** 1 Center of Innovation for Long-Term Services and Supports, Providence Veterans Affairs Medical Center, Providence, Rhode Island; 2 Brown University School of Public Health, Providence, Rhode Island; 3 Hartford Hospital, Hartford, Connecticut; 4 National Social Work Program, Care Management and Social Work Services, Patient Care Services, Department of Veterans Affairs, Washington, District of Columbia; 5 Northport VA Medical Center, Northport, New York; 6 Tomah VA Medical Center, Tomah, Wisconsin; 7 VA Tennessee Valley Healthcare System, Nashville

## Abstract

**Importance:**

Palliative care improves quality of life for patients and families but may be underused.

**Objective:**

To assess the association of an intervention to increase social work staffing in Veterans Health Administration primary care teams with use of palliative care among veterans with a recent hospitalization.

**Design, Setting, and Participants:**

This cohort study used differences-in-differences analyses of the change in palliative care use associated with implementation of the Social Work Patient Aligned Care Team (PACT) staffing program, conducted from October 1, 2016, to September 30, 2019. The study included 71 VA primary care sites serving rural veterans. Participants were adult veterans who received primary care services from a site enrolled in the program and who received inpatient hospital care. Data were analyzed from January 2020 to August 2022.

**Exposures:**

The PACT staffing program was a clinic-level intervention that provided 3-year seed funding to Veterans Health Administration medical centers to hire 1 or more additional social workers in primary care teams. Staggered timing of the intervention enabled comparison of mean outcomes across sites before and after the intervention.

**Main Outcomes and Measures:**

The primary outcome was the number of individuals per 1000 veterans who had any palliative care use in 30 days after an inpatient hospital stay.

**Results:**

The analytic sample included 43 200 veterans (mean [SD] age, 65.34 [13.95] years; 37 259 [86.25%] men) and a total of 91 675 episodes of inpatient hospital care. Among the total cohort, 8611 veterans (9.39%) were Black, 77 069 veterans (84.07%) were White, and 2679 veterans (2.92%) were another race (including American Indian or Alaskan Native, Asian, and Native Hawaiian or other Pacific Islander). A mean of 14.5 individuals per 1000 veterans (1329 individuals in all) used palliative care after a hospital stay. After the intervention, there was an increase of 15.6 (95% CI, 9.2-22.3) individuals per 1000 veterans using palliative or hospice care after a hospital stay, controlling for national time trends and veteran characteristics—a 2-fold difference relative to the mean.

**Conclusions and Relevance:**

This cohort study found significant increases in use of palliative care for recently hospitalized veterans whose primary care team had additional social work staffing. These findings suggest that social workers may increase access to and/or use of palliative care. Future work should assess the mechanism for this association and whether the increase in palliative care is associated with other health or health care outcomes.

## Key Points

**Question:**

Is additional social work staffing in primary care associated with a higher use of palliative care among recently hospitalized patients?

**Findings:**

In this cohort study involving 43 200 veterans in the Department of Veterans Affairs health system, the addition of social workers to primary care teams was associated with nearly doubled use of palliative care among veterans who had had inpatient hospital care compared with before the increase in social work staffing.

**Meaning:**

These findings suggest that social workers in primary care teams may facilitate access to palliative care for recently hospitalized patients.

## Teaser

This cohort study assesses the association of an intervention to increase social work staffing in Veterans Health Administration primary care teams with use of palliative care among veterans with a recent hospitalization.

## Introduction

Palliative care improves outcomes for patients and their caregivers.^1,2,3,4,5^ Within the Department of Veterans Affairs (VA) health system, veterans can receive palliative and hospice care concurrently with other medical treatments,^6^ and systemwide initiatives in the early 2000s expanded access to palliative and end-of-life services.^7,8^ However, individuals who are eligible for palliative care often do not receive it.^9^ Patients who undergo surgery, for example, are less likely to receive palliative care than their counterparts who receive medical care.^10^ Veterans may not use palliative care because they do not understand what it is or its availability or because clinicians are reluctant to make referrals.^11,12^ Access to palliative care may be particularly limited in rural areas.^13^ Given the advantages of palliative care to patients and health systems, health care leaders stand to benefit from a better understanding of what interventions can expand use of palliative care services.

Health care social workers have the skills to overcome barriers to palliative care, including educating patients, helping them understand their options, and eliciting preferences for care. We examined the association of social workers with use of palliative care through an evaluation of a program to embed social workers in VA Patient Aligned Care Teams (PACTs). The Social Work PACT staffing program provided funding to create new social work positions for primary care teams. We focused on veterans with recent hospital stays because hospitalization is a sentinel event in the life course of older patients that often marks a decline in health or change in disease trajectory. The decreased recovery may be subclinical or symptomatic but is a potential trigger for palliative care. From the perspective of the social work service, hospitalization is often a time when the conversation about palliative care starts. Social workers can bridge the transition from inpatient care to outpatient, when patients return to the prehospitalization environment with new appointments, medications, and therapies. We hypothesized that after an increase in social work staffing in the outpatient setting, we would observe increased use of palliative care among the clinic’s patients who were returning from hospital stays.

## Methods

### Study Design

This cohort study was conducted as part of an evaluation comprising quality-improvement activities on behalf of the VA National Social Work Program Office; therefore, the Providence VA Medical Center institutional review board determined the study exempt from review and informed consent. We conducted a retrospective cohort study documenting the impact of the Social Work PACT Staffing Program on palliative care and hospice utilization among veterans enrolled in primary care at a site that participated in the staffing program. We followed the Strengthening the Reporting of Observational Studies in Epidemiology (STROBE) reporting guidelines for cohort studies.

### Study Setting, Data, and Participants

During the study period between October 1, 2016, and September 30, 2019, 71 primary care clinics at VA medical centers entered the Social Work PACT staffing program (eTable 1 in Supplement 1). We identified all veterans who had at least 1 primary care visit at a participating site at any point in the study period to assign a veteran to an outpatient clinic. If a veteran had primary care at more than 1 clinic, we assigned them to the site where they had the most visits or the most recent visit in case of a tie. Among these, we included in our cohort veterans who had at least 1 inpatient medical or surgical hospital stay. Veterans with more than 1 inpatient admission are observed multiple times in our data, including readmissions within 30 days.

### Intervention

The Social Work PACT staffing program funded PACT social work positions for 3-year terms at sites serving rural veterans. The program funded social workers at outpatient clinics colocated in a medical center as well as stand-alone, community-based outpatient clinics. The *VA PACT Handbook*^14^ suggests an optimal 1 social worker per 2 advanced-practice clinicians, that is, a panel of 2400 veterans per social worker. To be eligible for program funding, the clinics had fewer than the recommended ratio and served a substantial number of rural veterans. The program funded the social worker positions for the first 3 years, after which the medical center could choose to fund the position from its local budget. Social workers were trained in the VA Social Work Practice Model, which emphasizes comprehensive assessment of patients’ psychosocial needs followed by ongoing case management and treatment planning. The program intervention has previously been found to be associated with reductions in emergency department visits and hospitalizations among veterans at high risk of of hospitalization or mortality.^15^

### Outcomes

We operationalized the outcome as the number of individuals per 1000 veterans who had any palliative care in the 30-day period after an acute inpatient hospital stay at either a VA hospital or VA-paid stay at a community hospital. All palliative care encounters and in the VA and VA-paid care in community settings were included. Because VA clinicians use palliative and hospice codes interchangeably when documenting care encounters, we included VA hospice encounters in the outcome but not enrollment in a home hospice program, nursing home hospice center, or Medicare-paid hospice.

### Statistical Analysis

We used linear regression and a difference-in-difference approach to measure in the change in palliative care use after the intervention, compared with baseline. The key explanatory variable of interest was an indicator for when a site hired its first social worker using program funds. The analysis was intent-to-treat, that is, we estimated average treatment effects among all veterans whose primary care team was actively participating in the social work staffing program at the time that of the veteran’s hospital stay, regardless of whether they had a social work encounter. To allow for a training period for new social workers, we excluded observations from a site in the first 2 months of the first social worker’s start date. The program had staggered start dates because additional funds became available over time and because of idiosyncratic variation in the timing for recruitment, hiring, and onboarding of new social workers. This staggered intervention allowed us to adjust for both site fixed–effects, which account for time-constant differences among the sites, and time fixed–effects, which adjust for secular changes in use of palliative care across the VA. We also adjusted for individual demographics (ie, age [linear and 5-year bins], race, gender, rural home residence), chronic diagnoses within the previous 12 months (congestive heart failure, hypertension, diabetes, tumor, lymphoma, metastatic cancer, dementia, psychiatric diagnoses, substance use disorder, and the count of the number of conditions from the Elixhauser index), and behavioral and social risk factors (current smoker or unstably housed or homeless). Race was self-reported or, for individuals unable to self-report, by a proxy (caregiver), VA enrollment coordinator, or clerk. We grouped race categories into Black, White, and other (eg, American Indian or Alaskan Native, Asian, or Native Hawaiian or other Pacific Islander) and missing (decline to answer, unknown, or missing). We adjusted for race because of previously documented racial disparities in use of VA care.^16^ A full descriptive table of variables as entered in the model is provided in eTable 2 in Supplement 1. To assess the similarity of the populations before and after the intervention, we examined characteristics before and after the intervention (eTable 3 in Supplement 1).We report the results from both 2-way fixed-effects estimators and doubly robust estimators in which only not-yet-treated groups serve as controls^17^ We evaluated parallel trends in the outcome prior to the intervention with a dynamic event-study plot (eFigure in Supplement 1). Because veterans who receive hospital care outside of the VA system may have differences in their access to palliative care, we performed a sensitivity analysis in which we stratified the cohort into patients with only VA inpatient days and patients who had 1 or more days of care from a community hospital. To examine whether use in palliative care services may have been associated with changes in the inpatient setting, rather than outpatient primary care teams, we analyzed palliative care specialist consultations ordered during the hospital stay. We report additional information on model specification and estimators in the eAppendix in Supplement 1.

Statistical analyses were performed using R software version 3.6.1 (R Project for Statistical Computing). To compare the VA-only and community hospital samples, we report standardized mean differences and *P* values of difference in means from a 2-sided *t* test. In regression models, we clustered SEs by site, as this was the level of the intervention.^18^ That clustering approach also corrected the SEs for correlation among repeated observations from veterans since in our data, veterans were nested within primary care sites. We assessed statistical significance of model coefficients using 95% CIs. We calculated 95% coverage intervals for the doubly-robust estimates using 1000 bootstrap iterations. *P *values were 2-sided, but we did not prespecify a threshold for statistical significance. Data were analyzed from January 2020 to August 2022.

## Results

the final sample included 43 200 veterans (mean [SD] age, 65.34 [13.95] years; 37 259 [86.25%] men) who contributed a total of 91 675 episodes of care to the data set from October 2016 to September 2019. Of these, 32 704 veterans only had stays at a VA hospital, and 17 347 veterans also had stays at community hospitals (Table 1). Among the total cohort, 8611 veterans (9.39%) were Black, 77 069 veterans (84.07%) were White, and 2679 veterans (2.92%) were another race (including American Indian or Alaskan Native, Asian, and Native Hawaiian or other Pacific Islander). There were 35 571 veterans (38.80%) classified as having rural residence, and 16 031 veterans (17.49%) were unstably housed or experiencing homelessness. The most prevalent diagnoses from the previous 12 months included hypertension (67 536 veterans [73.67%]), diabetes (34 452 veterans [37.58%]), congestive heart failure (24 204 veterans [26.40%]), tumor (14 961 veterans [16.32%]), metastatic cancer (4255 veterans [4.64%]), dementia (8743 veterans [9.54%]), and psychiatric diagnosis (51 585 veterans [56.27%]), and mean (SD) Elixhauser was 6.0 (3.29). The VA-only and community-hospital groups had statistically significant differences in means across nearly all characteristics, with substantial differences in homelessness and smoking status (Table 1). Overall, 7127 veterans had a PACT social work encounter in the month following their hospital stay (265 individuals per 1000 veterans), and 1329 veterans used palliative care after their hospital stay (14.5 individuals per 1000 veterans). Palliative care use was 14.8 individuals per 1000 veterans among those with only VA hospitalizations, and 13.8 individuals per 1000 veterans among those who received care in community hospitals.

**Table 1.** Characteristics of Veterans in Sample

Characteristic	Veterans, No. (%)			*P* value	SMD
	With any hospital stay (N = 43 200)	With VA hospital stays only (n = 30 804)	With community hospital stays (n = 17 347)^a^		
Veteran-months, No.	91 675	62 924	28 751	NA	NA
Age, mean (SD), y	65.34 (13.95)	65.85 (14.15)	64.22 (13.45)	<.001	0.118
Race					
Black	8611 (9.39)	5894 (9.37)	2717 (9.45)	.70	0.003
White	77 069 (84.07)	54 001 (85.82)	23 068 (80.23)	<.001	0.149
Other^b^	2679 (2.92)	1064 (1.69)	1615 (5.62)	<.001	0.210
Missing	3316 (3.62)	1965 (3.12)	1351 (4.70)	<.001	0.081
Gender					
Men	37 259 (86.25)	27 186 (88.25)	15 024 (86.60)		
Women	5941 (6.48)	3618 (5.75)	2323 (8.08)	<.001	0.092
Rural residence	35 571 (38.80)	23 579 (37.47)	11 992 (41.71)	<.001	0.087
Unstably housed or homeless^c^	16 031 (17.49)	12 425 (19.75)	3606 (12.54)	<.001	0.197
Chronic disease diagnosis^c^					
Congestive heart failure	24 204 (26.40)	16 197 (25.74)	8007 (27.85)	<.001	0.048
Hypertension	67 536 (73.67)	46 649 (74.14)	20 887 (72.65)	<.001	0.034
Diabetes	34 452 (37.58)	23 583 (37.48)	10 869 (37.80)	.35	0.007
Tumor	14 961 (16.32)	10 916 (17.35)	4045 (14.07)	<.001	0.090
Lymphoma	1719 (1.88)	1158 (1.84)	561 (1.95)	.26	0.008
Metastatic cancer	4255 (4.64)	3062 (4.87)	1193 (4.15)	<.001	0.035
Dementia	8743 (9.54)	6666 (10.59)	2077 (7.22)	<.001	0.118
Psychiatric diagnosis	51 585 (56.27)	36 490 (57.99)	15 095 (52.50)	<.001	0.111
Substance use disorder	24 660 (26.90)	18 153 (28.85)	6507 (22.63)	<.001	0.143
Current smoker^c^	39 210 (42.77)	29 577 (47.00)	9633 (33.50)	<.001	0.278
Elixhauser score, mean (SD)	6.02 (3.29)	6.16 (3.26)	5.72 (3.34)	<.001	0.133
Veterans with any PACT social work after hospitalization, No. (No. per 1000 veterans)	7127 (265)	4382 (234)	2745 (337)	<.001	0.229
Veterans with any palliative care after hospitalization, No. (No. per 1000 veterans)	1329 (14.5)	932 (14.8)	397 (13.8)	.25	0.008

Abbreviations: NA, not applicable; PACT, Patient Aligned Care Team; SMD, standardized mean difference; VA, Department of Veterans Affairs.

^a^ VA-paid hospital stay at a non-VA hospital. This group includes some veterans who also had VA hospital use.

^b^ Other race includes American Indian or Alaskan Native, Asian, and Native Hawaiian or other Pacific Islander.

^c^ In 12 months prior to index month.

Table 2 shows the change in individuals who received palliative care per 1000 veterans that was associated with implementation of the Social Work PACT staffing program, adjusting for year, site, and veteran characteristics. After the program intervention, use of palliative care increased by 5.3 (95% CI, 2.3-8.3) individuals per 1000 veterans (*P* = .001), a 34% increase compared with the overall mean. Among veterans with only VA hospital care, 5.3 (95% CI, 1.4-9.1) additional individuals used palliative care per 1000 veterans (*P* = .009), representing a 39% increase from the overall means. Among veterans with community hospital care, an additional 6.0 (95% CI, −0.2 to 12.1) individuals used palliative care per 1000 veterans, although the difference was not statistically significant (*P* = .06). Using a double-robust estimator that used only not-yet-treated sites as the comparison group, the estimates were larger: 15.6 (95% CI, 9.2-22.3) additional individuals per 1000 veterans in the sample overall, 14.3 (95% CI, 5.0-22.7) additional individuals per 1000 veterans in the VA-only group, and 10.4 (95% CI, 4.1-16.8) additional individuals per 1000 veterans in the community-hospital group. These estimates represent more than 2-fold increases in palliative care use. Similar results were found when we used different weighting methods for this estimator (eTable 4 and eTable 5 in Supplement 1). In our falsification test, we did not detect a statistically significant association of the program with inpatient palliative care consultations during a veteran’s hospital stay (eTable 6 in Supplement 1).

**Table 2.** Association of an Additional Social Worker With Individuals per 1000 Veterans Who Received Palliative Care or Hospice Services in the Month After a Hospital Stay by Type of Hospitalization, October 2016 to September 2019

Model	Change, No. per 1000 veterans (95% CI)		
	Any hospitalization	VA hospitalization	Community hospitalization
2-Way fixed-effects model^a^	5.3 (2.3 to 8.3)	5.3 (1.4 to 9.1)	6.0 (−0.2 to 12.1)
*P* value	.001	.009	.06
Double-robust (group-time average)^b^	15.6 (9.2 to 22)	14.3 (5.9 to 22.7)	10.4 (4.1 to 16.8)

^a^ Adjusted for site and year-month fixed effects and demographic and clinical characteristics listed in Table 1.

^b^ Change in outcome for intervention sites is compared with not-yet-treated sites. Association is estimated separately for each group of sites that received the intervention in the same month, and these estimates are combined in a weighted mean based on the size of each group.

## Discussion

This cohort study found that after the addition of social workers, VA primary care teams saw statistically significant and clinically important increases in use of palliative care among veterans with recent inpatient hospital care compared with before the social workers were hired. Geographic, knowledge, and health system barriers may hinder veterans’ access to palliative and hospice care.^19^ Primary care practitioners (ie, physicians, nurse practitioners, physician assistants) report they have inadequate preparation for psychosocial care of patients who are terminally ill or are unsure of how or when to refer patients to hospice or palliative care services.^20,21^ Palliative care use is lower among patients in rural areas,^22^ while it is also particularly cost-effective in rural settings.^23^

The roles of social workers in primary care suggest several ways that PACT social workers could facilitate veterans’ use of palliative care. PACT social workers have a high level of competency in facilitating advanced directives, intervening in situations of family or caregiver distress, and addressing diverse cultural and spiritual needs.^24^ Thus, PACT social workers may play a role in recognizing caregiver stress or psychosocial barriers to access and facilitating referrals to palliative care clinics and specialists. For example, a patient may not yet be ready to consider palliative care while in the hospital, but in the outpatient setting, a PACT social worker might start the conversation about a shift to palliative or hospice care. Additionally, social workers may coordinate palliative care services directly at the time that the patient is ready to use them. Alternatively, social workers can intervene longitudinally, by educating a patient about palliative services and helping them with advanced care planning.^25^ These conversations could occur at multiple points in the care journey, before or after a decline in health leads to hospitalization. Advanced care preferences documented earlier in the disease course can alert the hospital team to the patient’s wishes for palliative measures and allow the patient and family or caregiver to advocate more strongly for their preferences in the inpatient setting. Social workers frequently address patients’ end-of-life needs, such as psychological and emotional care of family members, caregiver involvement, and care coordination.^26^ Specifically, veterans who have had a recent hospitalization may be prepared to update their goals of care, and PACT social workers can provide those resources and education. PACT social workers could also engender more palliative care referrals through culture change, team meetings regarding complex patients, and clinical education. For example, a social worker advocating or arranging palliative care for one patient could have spillover effects for subsequent patients by routinizing screening, referral networks, and other processes for not just social work staff but also care transition nurses, physician assistants, and primary care physicians.

Our findings suggest further steps for policy, practice, and research. System-wide improvements in the VA, such as training for clinicians in goals-of-care discussions and standardized documentation in the VA health record, have resulted in increased availability and access to palliative care.^27^ As health system leaders consider where to invest resources, social workers present a patient-centered approach to increase the use of a service that can improve outcomes and decrease readmissions.^28^ Increasing access to palliative and hospice care may partly explain how primary care social work staffing can decrease unplanned emergency and inpatient care.^15^ Further research should examine the effects that increasing palliative care use through social work staffing can have on patients’ health outcomes and health care use.

The strengths of this study are that it is an evaluation of a national program involving many sites, broad geographic distribution, and a large sample of patients. Although the intervention was not randomized, the variation in the timing of the program across sites allowed for a study design that controlled for many potential confounders, including differences among sites and secular time trends. We tested the association of initiation of the Social Work PACT staffing program with palliative care consultations, which are usually ordered by the hospitalist team. We found no association with consultations, which is consistent with a direct outcome of the Social Work PACT staffing program rather than other, simultaneous changes to medical center operations.

### Limitations

This study has some limitations. First, we were not able to rule out other health system interventions related to palliative care that may have coincided with each site’s participation in the Social Work PACT staffing program. With the available data, we were able to measure only care provided by or paid for by the VA and did not include care through Medicare or other private insurance. Our findings may not be generalizable. VA medical centers that participated in the Social Work PACT staffing program were not representative of VA primary care clinics nationally, in that they were understaffed with respect to the recommended number of social workers and served more veterans from rural areas. Further research should compare palliative care use and social work staffing ratios between participating sites and nonparticipating sites. Furthermore, our findings may not generalize to health systems with different services and eligibility for benefits. For example, Medicare’s hospice benefit, which can provide home health aides and respite care, is only available to patients who forego curative treatment.^29^

## Conclusions

The findings of this cohort study suggest that adding a social worker to the primary care team may help veterans to access palliative care. The findings from this program evaluation offer insights into how investment in social workers may increase use of a high-value service.

## Notes

Supplement 1. **eAppendix.** Supplementary Methods **eTable 1.** Intervention Sites, Starting Month, and Facility Characteristics **eTable 2.** Descriptive Table of Variables Used Subsequent Models and Other Demographics of Interest **eTable 3.** Characteristics of Hospitalized Veterans Before and After Intervention **eTable 4.** Sensitivity Analyses: Program Outcome Using Different Estimators **eTable 5.** Effects Grouped by Sites That Started the Staffing Program in the Same Month **eTable 6.** Falsification Tests Using Inpatient Consults and Mortality as Outcomes **eFigure.** Event Study and Evaluation of Parallel Trends

Supplement 2.
Data Sharing Statement
