# Supplementary material for: Social Work Staffing and Use of Palliative Care Among Recently Hospitalized Veterans
Source: JAMA Netw Open. 2023 Jan 4;6(1):e2249731. doi: 10.1001/jamanetworkopen.2022.49731 (PMC9856777; doi:10.1001/jamanetworkopen.2022.49731)
Supplement: Supplement 1. — eAppendix. Supplementary Methods eTable 1. Intervention Sites, Starting Month, and Facility Characteristics eTable 2. Descriptive Table of Variables Used Subsequent Models and Other Demographics of Interest eTable 3. Characteristics of Hospitalized Veterans Before and After Intervention eTable 4. Sensitivity Analyses: Program Outcome Using Different Estimators eTable 5. Effects Grouped by Sites That Started the Staffing Program in the Same Month eTable 6. Falsification Tests Using Inpatient Consults and Mortality as Outcomes eFigure. Event Study and Evaluation of Parallel Trends [file jamanetwopen-e2249731-s001.pdf]

Supplemental Online Content

Cornell PY, Halladay CW, Montano AR, et al. Social work staffing and use of palliative care among recently hospitalized veterans. *JAMA Netw Open*. 2023;6(1):e2249731. doi:10.1001/jamanetworkopen.2022.49731

eAppendix. Supplementary Methods
eTable 1. Intervention Sites, Starting Month, and Facility Characteristics
eTable 2. Descriptive Table of Variables Used Subsequent Models and Other Demographics of Interest
eTable 3. Characteristics of Hospitalized Veterans Before and After Intervention
eTable 4. Sensitivity Analyses: Program Outcome Using Different Estimators
eTable 5. Effects Grouped by Sites That Started the Staffing Program in the Same Month
eTable 6. Falsification Tests Using Inpatient Consults and Mortality as Outcomes
eFigure. Event Study and Evaluation of Parallel Trends

This supplemental material has been provided by the authors to give readers additional information about their work.

© 2023 Cornell PY et al. *JAMA Network Open.*

eAppendix. Supplementary Methods

Data and measures

Sample and setting

eTable 1 lists the VA sites that began the staffing program in our study period. Of the 71 sites, 40 (75%) were community-based outpatient clinics (CBOCs) or multi-specialty CBOCs, 10 (14%) were located at a VA medical center (hospital), and 8 (10%) were other outpatient services facilities or health care centers. Veterans can access some palliative services from their primary care clinician or VA-paid community hospice and palliative services, and all VA medical centers have specialty inpatient palliative services on site.

We assigned Veterans to a site based on where they had the most primary care encounters in a fiscal year. Each Veteran's baseline characteristics and outcomes are measured from an index date in the calendar month. If a Veteran had a primary care encounter in that month, the first primary care encounter is the index date; if they did not have a primary care encounter, their index date is assigned to the 15th of the month. For this analysis, we included Veterans who had any inpatient hospital (VA or community) care within the 30 days prior to the index date.

Outcome

The palliative care outcome includes encounters with the VA "stop" codes 351 or 353 in the first or second position; and inpatient specialty codes ("bed section codes") 96 and 1F. These codes are used interchangeably by providers for hospice and palliative care. In billing records, we identified community-provided services with the following "hospice/palliative care" purpose-of-visit codes: 77, 78, 43, 37, and 38.

VA policy mandates that specialty care occurs within 28 days of the order; so, if an order for outpatient care is placed at discharge, we would expect to meet the 30-day mark because of scheduling mandates. Alternatively, we suppose that Veterans would have a transition nurse phone call within 2 days of discharge; a follow-up PACT appointment, if needed, would be scheduled within two weeks; and if needs are identified at that appointment, another 2 weeks for referral and palliative care services to be delivered. Thus we deemed 30 days a reasonable interval to accommodate clinical variation and capture most palliative care services set in motion by transitions in care.

Exposure

Exposure to the program is measured by the month at the site level. The intervention date is the first month in which a social worker is hired to a program-funded position. Some sites received funding for multiple full-time-equivalent positions, and some individuals covered multiple smaller clinics in their position. We excluded sites' data for the first and second month of the new social work position to allow for a training period.

Estimation strategies

Two-way fixed effects

The standard approach to estimating an intervention effect with variation in treatment timing is the two-way fixed-effects (TWFE) linear regression. This is a linear regression as follows:

$$Y_{it} = \alpha D_{st} + \theta_t + \eta_s + \gamma X_{ist} + \nu_{ist}$$

where θ_t is a year-month fixed effect, η_s is fixed effect for sites (i.e., primary care clinic or community-based outpatient clinic), D_{is} is a dummy variable representing participation in the program at time t, X_{ist} are individual Veteran characteristics, ν_{ist} are time varying unobservable variables that are mean independent of everything else, and α is the effect of the staffing intervention. This approach is a standard in applied work and historically was considered a strong quasi-experimental design because η_s controls for systematic differences between Veterans at sites that are consistent over time and θ_t controls for secular changes over time.
In our sample, all sites eventually participate in the program. Therefore, in our TWFE estimators newly treated sites are compared to "not yet treated" sites and "already treated" sites. It requires some key assumptions, including that 1) there are no exogenous events other than the program that affect the outcome and coincide with the treatment intervention, and 2) the trends in outcome for treatment and comparison groups would be similar, or parallel, in the absence of the intervention. In this study, the staggered timing of the program start dates decrease the likelihood of a time-varying confounder that coincided with the intervention. We address the second parallel trends assumption by examining pre-trends in the outcome (see below).

Difference-in-difference: alternative estimators
Recent work has shown that the traditional TWFE estimators can result in hard-to-interpret weights and even negative weights for some units because of dynamic treatment effects.[1] To address this problem, we also estimate our program impact using Sant'Anna and Callaway's approach with group-time-average treatment effects. Sites are grouped by the month when they started the staffing program. The effect of the program is estimated for each group, using the not-yet-treated sites as the comparison sample. We report the aggregated group-time-averaged effects: the program effect is calculated for each group of clinics that began the intervention at the same time, and these effects are then weighted by the size of the groups.

Sensitivity analyses and checks
Parallel trends assumption
We created and event study plot using the DID package to examine trends in the outcome relative to the comparison in the months leading up to treatment (figure S1). The pre-treatment dummy coefficients are statistically not different from zero, and suggest that prior to the intervention the trend in palliative care may, if anything, trend downward relative to the trend of the comparison sites.

Collider bias: sample selection after the social work staffing intervention
Previous work has shown that the VA Social Work Staffing Program may decrease the risk of hospitalization for high-risk Veterans, which could lead to "collider bias" because the intervention could be associated with selection into the sample in ways that are systematically correlated with use of palliative care.[2] While we cannot entirely rule out possible collider bias, we show that the characteristics of Veterans hospitalized in the month before and two months after the intervention are substantially similar (**Error! Reference source not found.**). Because o ur study cohort is an unbalanced panel, the table shows only hospitalizations in the month before and the third month after the intervention. The table shows only very small differences between the groups for the demographics and comorbidities in our sample, with all SMD<0.05 (a

© 2023 Cornell PY et al. *JAMA Network Open.*

conservative rule of thumb for covariate balance is that variables have SMD<0.1). Although this check does not rule out the possibility that selection on unmeasured characteristics could bias selection into hospitalization, it is suggestive that systematic differences before and after intervention in the characteristics of hospitalized Veterans are unlikely.

Nonetheless, it is useful to explore the likely extent and direction of collider bias from previous work. In previous work, we found that the social work program intervention was associated with a decrease of approximately 4% decrease in hospitalizations among high-risk Veterans, and no significant effect among the population at large. If those "averted" hospitalizations were among Veterans that would have had low or no palliative care use, the sample difference in the denominator could bias our estimate of the program's effect on palliative care in the positive direction. In a back-of-the-envelope example, suppose we have a group of 1000 hospitalized Veterans, 14.5 who use palliative care, prior to program intervention. After the social work intervention, this group would be reduced by 4.4%, so the new denominator is =956. In the absence of any true program effect on palliative care use, the new rate is 14.5/956 = 15.2. This example puts an upper bound of 0.7 Veterans per 1000 that we are over-estimating due to collider bias. Because the lower bound of the 95% confidence intervals were >2 per 1000 for the overall sample, even accounting for this bias we would still see an impact of the social work program on primary care.

Competing risk: Check for change in 30-day mortality associated with the social work program
Increased survival and time at risk could explain an increase in palliative care referrals. We checked for a change in mortality associated with the social work staffing intervention (**Error! Reference source not found.**). We found small and not statistically significant associations of the intervention with 30-day mortality, suggesting that our results were unlikely to be driven by changes in survival.

Falsification test with palliative care inpatient consults
Because it is possible that other changes in health system delivery that were correlated over time with the staffing intervention may have influenced use of palliative care, we investigated the association of inpatient palliative care consults with the intervention (**Error! Reference source not found.**). We identified consults through orders in the health record with associated stop codes of 351 or 353.

Supplement References

1. Callaway B, Sant'Anna PHC. Difference-in-Differences with multiple time periods. Journal of Econometrics. 2021;225(2):200–30.
2. Griffith GJ, Morris TT, Tudball MJ, Herbert A, Mancano G, Pike L, et al. Collider bias undermines our understanding of COVID-19 disease risk and severity. Nature communications. 2020;11(1):1-12.

© 2023 Cornell PY et al. *JAMA Network Open.*

eTable 1. Intervention Sites, Starting Month, and Facility Characteristics

Station ID	Division Name	First Treat Mnth	State	ZIP3	VISN	Type	Provider based
517GB	GREENBRIER CBOC	2016-11	WV	249	5	CBOC	PBO
517QA	PRINCETON VA CLINIC	2016-11	WV	247	5	OOS	PBO
519GA	ODESSA CBOC	2016-11	TX	797	17	MSCBOC	PBO
519GB	HOBBS	2016-11	NM	882	17	CBOC	PBO
519GD	FT STOCKTON	2016-11	TX	797	17	OOS	PBO
541GJ	NEW PHILADELPHIA	2016-11	OH	446	10	MSCBOC	PBO
517	BECKLEY VAMC	2017-01	WV	258	5	VAMC	PBH
528G9	CORTLAND CBOC	2017-01	NY	130	2	CBOC	PBO
528GM	ROME,NY	2017-01	NY	134	2	MSCBOC	PBO
528GN	BINGHAMTON,NY	2017-01	NY	139	2	MSCBOC	PBO
589A6	LEAVENWORTH VAMC DIVISION	2017-01	KS	660	15	VAMC	PBH
657GK	MT. VERNON CBOC-MA	2017-01	IL	628	15	CBOC	PBO
657GU	HARRISBURG CBOC-MA	2017-01	IL	629	15	CBOC	PBO
528A7	SYRACUSE,NY	2017-02	NY	132	2	VAMC	PBH
528G5	AUBURN CBOC	2017-02	NY	130	2	CBOC	PBO
528GO	WATERTOWN CBOC	2017-02	NY	136	2	MSCBOC	PBO
528GP	OSWEGO,NY	2017-02	NY	131	2	CBOC	PBO
541GC	SANDUSKY	2017-02	OH	448	10	MSCBOC	PBO
549BY	FORT WORTH OPC	2017-02	TX	761	17	MSCBOC	PBO
549GE	BRIDGEPORT VA CLINIC	2017-02	TX	762	17	CBOC	PBO
589A5	TOPEKA VAMC DIVISION	2017-02	KS	666	15	VAMC	PBH
610A4	FORT WAYNE, IN DIVISION	2017-02	IN	468	10	VAMC	PBH
610GC	GOSHEN CBOC	2017-02	IN	465	10	CBOC	NPB
657GM	EFFINGHAM CBOC-MA	2017-02	IL	624	15	CBOC	PBO
436	FT HARRISON MT	2017-03	MT	596	19	VAMC	PBH
528G3	BAINBRIDGE CBOC	2017-03	NY	137	2	CBOC	PBO
528G6	FONDA CBOC	2017-03	NY	120	2	CBOC	PBO
528G7	CATSKILL CBOC	2017-03	NY	124	2	CBOC	PBO
541GH	EAST LIVERPOOL	2017-03	OH	439	10	MSCBOC	PBO
549A4	BONHAM	2017-03	TX	754	17	DOM	PBH
549GH	GREENVILLE CBOC	2017-03	TX	754	17	CBOC	PBO
549GJ	SHERMAN CBOC	2017-03	TX	750	17	CBOC	NPB
610	MARION, IN DIVISION	2017-03	IN	469	10	VAMC	PBH
583GB	BLOOMINGTON CBOC	2017-04	IN	474	10	CBOC	PBO
610GD	PERU CBOC	2017-04	IN	469	10	CBOC	PBO
515GC	BENTON HARBOR	2017-05	MI	490	10	CBOC	PBO
583GC	MARTINSVILLE CBOC	2017-05	IN	461	10	CBOC	PBO
583GF	WAKEMAN VA CLINIC	2017-05	IN	461	10	MSCBOC	PBO
589GR	JUNCTION CITY CBOC - EKH	2017-05	KS	664	15	CBOC	PBO
575	GRAND JUNCTION	2017-06	CO	815	19	VAMC	PBH
575GA	CBOC-MONTROSE	2017-06	CO	814	19	OOS	PBO
575QB	MOAB VA CLINIC	2017-06	UT	845	19	OOS	NPB
589GI	ST. JOSEPH CBOC - EKH	2017-06	MO	645	15	CBOC	PBO
575GB	CRAIG TELEHEALTH OUTRCH CLINIC	2017-07	CO	816	19	OOS	PBO
529GB	LAWRENCE COUNTY	2017-08	PA	161	4	CBOC	PBO
583GA	TERRE HAUTE CBOC	2017-08	IN	478	10	CBOC	PBO
459GC	KONA CLINIC	2017-09	HI	967	21	CBOC	PBO
460GA	MILLSBORO	2017-10	DE	199	4	CBOC	PBO
607GG	MADISON WEST VA CLINIC	2017-10	WI	537	12	CBOC	PBO
459GA	MAUI CLINIC	2017-11	HI	967	21	CBOC	PBO
459GE	GUAM CLINIC	2017-12	GU	969	21	CBOC	NPB
529	BUTLER VAMC	2017-12	PA	160	4	VAMC	PBH
676GD	WISCONSIN RAPIDS SATELLITE	2017-12	WI	544	12	MSCBOC	PBO
541GD	MANSFIELD	2018-01	OH	449	10	MSCBOC	PBO
676GA	WAUSAU SATELLITE	2018-01	WI	544	12	CBOC	PBO
676GE	CLARK COUNTY SATELLITE	2018-01	WI	544	12	CBOC	PBO

© 2023 Cornell PY et al. *JAMA Network Open.*

Station ID	Division Name	First Treat Mnth	State	ZIP3	VISN	Type	Provider based
459GB	HILO CLINIC	2018-02	HI	967	21	CBOC	PBO
459GF	AMERICAN SAMOA CLINIC	2018-02	AS	967	21	CBOC	PBO
459GH	SAIPAN VA CLINIC	2018-02	MP	969	21	OOS	NPB
459QB	MOLOKAI VA CLINIC	2018-02	HI	967	21	OOS	NPB
607GD	BARABOO CBOC	2018-02	WI	539	12	CBOC	PBO
607GE	BEAVER DAM CBOC	2018-02	WI	539	12	CBOC	PBO
607GF	FREEPORT CBOC	2018-02	IL	610	12	CBOC	PBO
607GC	JANESVILLE CBOC	2018-03	WI	535	12	CBOC	PBO
460HE	VENTNOR	2018-04	NJ	82	4	MSCBOC	NPB
460HG	VINELAND	2018-04	NJ	83	4	MSCBOC	PBO
553GA	YALE CBOC	2018-04	MI	480	10	CBOC	PBO
553GB	PONTIAC CBOC	2018-04	MI	483	10	CBOC	PBO
676GC	LACROSSE SATELLITE	2018-05	WI	546	12	CBOC	PBO
676	TOMAH	2018-06	WI	546	12	VAMC	PBH
506GC	JACKSON OUTPATIENT CLINIC	2019-10	MI	492	10	CBOC	PBO

Abbreviations
CBOC = Primary Care Community-Based Outpatient Clinic
MSCBOC = Multi-Specialty Community Based Outpatient Clinic
CLC = Community Living Center located on VAMC Ward/Floor
SACLC - Stand Alone Community Living Center
CMC = Civilian Medical Center
DES= Disability Evaluation Service Clinic
SADOM = Stand Alone Domiciliary
DOM = Domiciliary located on VAMC Ward/Floor
HCC= Health Care Center
MHRRTP = Mental Health Residential Rehab Treatment Program
NPB = Non-Provider-Based Outpatient Facility
OOS = Other Outpatient Services Facility
PB = Provider-Based/Non-Provider-Based Designation
PBH = Provider-Based Hospital
PBO = Provider-Based Outpatient Facility
PRRTP = Psychosocial Residential Rehab Treatment Program
SRRTP = Substance Residential Rehab Treatment Program
VAMC = VA Medical Center
Sta # = VA Facility Station Number
ST = State
VN = Veterans Integrated Service Network
ZIP 3 = First three digits of ZIP Code

© 2023 Cornell PY et al. *JAMA Network Open.*

eTable 2. Descriptive Table of Variables Used Subsequent Models and Other Demographics of Interest

	Veteran-months with hospital stays (in the previous month)	Veteran-months with VA hospital stays (in the previous month)	Veteran-months with community hospital stays (in the previous month)
Veterans, n	43,200	30,804	17,347
Veteran-months, n (%)	91,675 (100.00%)	62,924 (100.00%)	28,751 (100.00%)
Age at PC encounter, mean (SD)	65.34 (13.95)	65.85 (14.15)	64.22 (13.45)
Age [18, 45), n (%)	8,249 (9.00%)	5,520 (8.77%)	2,729 (9.49%)
Age [45, 50), n (%)	3,140 (3.43%)	2,111 (3.35%)	1,029 (3.58%)
Age [50, 55), n (%)	4,707 (5.13%)	3,255 (5.17%)	1,452 (5.05%)
Age [55, 60), n (%)	8,600 (9.38%)	5,800 (9.22%)	2,800 (9.74%)
Age [60, 65), n (%)	12,668 (13.82%)	8,265 (13.13%)	4,403 (15.31%)
Age [65, 70), n (%)	17,471 (19.06%)	11,641 (18.50%)	5,830 (20.28%)
Age [70, 75), n (%)	17,142 (18.70%)	11,427 (18.16%)	5,715 (19.88%)
Age [75, 80), n (%)	7,401 (8.07%)	5,344 (8.49%)	2,057 (7.15%)
Age [80, 85), n (%)	5,211 (5.68%)	3,951 (6.28%)	1,260 (4.38%)
Age [85, 90), n (%)	4,230 (4.61%)	3,321 (5.28%)	909 (3.16%)
Age [90, 95), n (%)	2,226 (2.43%)	1,794 (2.85%)	432 (1.50%)
Age [95, 120), n (%)	630 (0.69%)	495 (0.79%)	135 (0.47%)
Race: Black, n (%)	8,611 (9.39)	5,894 (9.37)	2,717 (9.45)
Race: Other, n (%)	2,679 (2.92)	1,064 (1.69)	1,615 (5.62)
Race: Missing, n (%)	3,316 (3.62)	1,965 (3.12)	1,351 (4.70)
Gender: Women, n (%)	5,941 (6.48%)	3,618 (5.75%)	2,323 (8.08%)
Rural, n (%)	35,571 (38.80%)	23,579 (37.47%)	11,992 (41.71%)
CHF*, n (%)	24,204 (26.40%)	16,197 (25.74%)	8,007 (27.85%)
HTN*, n (%)	67,536 (73.67%)	46,649 (74.14%)	20,887 (72.65%)
Diabetes*, n (%)	34,452 (37.58%)	23,583 (37.48%)	10,869 (37.80%)

© 2023 Cornell PY et al. *JAMA Network Open.*

	Veteran-months with hospital stays (in the previous month)	Veteran-months with VA hospital stays (in the previous month)	Veteran-months with community hospital stays (in the previous month)
Tumor*, n (%)	14,961 (16.32%)	10,916 (17.35%)	4,045 (14.07%)
Lymphoma*, n (%)	1,719 (1.88%)	1,158 (1.84%)	561 (1.95%)
Metastatic Cancer*, n (%)	4,255 (4.64%)	3,062 (4.87%)	1,193 (4.15%)
Dementia*, n (%)	8,743 (9.54%)	6,666 (10.59%)	2,077 (7.22%)
Psych Diagnosis*, n (%)	51,585 (56.27%)	36,490 (57.99%)	15,095 (52.50%)
Substance Use Disorder*, n (%)	24,660 (26.90%)	18,153 (28.85%)	6,507 (22.63%)
Current Smoker*, n (%)	39,210 (42.77%)	29,577 (47.00%)	9,633 (33.50%)
Unstably Housed or Homeless*, n (%)	16,031 (17.49%)	12,425 (19.75%)	3,606 (12.54%)
Elixhauser Score*, mean (SD)	6.02 (3.29)	6.16 (3.26)	5.72 (3.34)
Active ORH-Funded Social Worker at Veteran's Site, n (%)	70,980 (77.43%)	48,343 (76.83%)	22,637 (78.73%)
1+ hospital days in previous 30 days, n (%)	91,675 (100.00%)	62,924 (100.00%)	28,751 (100.00%)
1+ VA hospital days in previous 30 days, n (%)	67,785 (73.94%)	62,924 (100.00%)	4,861 (16.91%)
1+ community hospital days in previous 30 days, n (%)	28,751 (31.36%)	0 (0.00%)	28,751 (100.00%)
1+ PACT SW encounter within 30 days after inpatient hospital stay	7,127 (26.51%)	4382 (23.39%)	2745 (33.68%)
# of Palliative Care encounters within 30 days, mean (SD)	0.09 (1.32)	0.08 (1.27)	0.10 (1.44)
1+ Palliative Care encounters within 30 days, n (per 1000)	1,329 (14.5 per 1000)	932 (14.8 per 1000)	397 (13.8 per 1000)

* Occurrence in the 365 days prior to palliative care encounter
Other race includes American Indian or Alaskan Native, Asian, and Native Hawaiian or other Pacific Islander

© 2023 Cornell PY et al. *JAMA Network Open.*

eTable 3. Characteristics of Hospitalized Veterans Before and After Intervention

	After Intervention	Before Intervention	Pr|H_0	SMD
n Veterans	2,755	2,553		
Age, mean (SD)	64.65 (14.39)	64.78 (14.50)	0.738	0.009
Race: Black, No. (%)	276 (10.02)	247 (9.67)	0.709	0.012
Race: White, No. (%)	84 (3.05)	86 (3.37)	0.56	0.018
Race: Other, No. (%)	2305 (83.67)	2123 (83.16)	0.645	0.014
Race: Missing, No (%)	90 (3.27)	97 (3.80)	0.328	0.029
Gender: female, No. (%)	186 (6.75)	154 (6.03)	0.311	0.029
Rural, No. (%)	1064 (38.62)	1045 (40.93)	0.091	0.047
Unstably housed or homeless[a], No. (%)	498 (18.08)	418 (16.37)	0.109	0.045
Congestive heart failure[a], No. (%)	731 (26.53)	669 (26.20)	0.81	0.007
Hypertension[a], No. (%)	2018 (73.25)	1897 (74.30)	0.399	0.024
Diabetes[a], No. (%)	1014 (36.81)	983 (38.50)	0.212	0.035
Tumor[a], No. (%)	454 (16.48)	383 (15.00)	0.151	0.041
Lymphoma[a], No. (%)	50 (1.81)	46 (1.80)	1	0.001
Metastatic cancer[a], No. (%)	122 (4.43)	105 (4.11)	0.617	0.016
Dementia[a], No. (%)	259 (9.40)	251 (9.83)	0.628	0.015
Psychiatric diagnosis[a], No. (%)	1544 (56.04)	1487 (58.25)	0.111	0.045
Substance use disorder[a], No. (%)	783 (28.42)	702 (27.50)	0.472	0.021
Current smoker[a], No. (%)	1238 (44.94)	1091 (42.73)	0.112	0.044
Elixhauser score, mean (SD)	5.95 (3.21)	5.94 (3.20)	0.936	0.002

Notes: "Before" includes the month prior to intervention start at sites, and "After" is the third month after the intervention start.
a. In 12 months prior to index month
b.VA-paid hospital stay at a non-VA provider
SMD=standardized mean difference

© 2023 Cornell PY et al. *JAMA Network Open.*

eTable 4. Sensitivity Analyses: Program Outcome Using Different Estimators

	Any Hospitalization		VA Hospitalization		Community Hospitalization	
Change in palliative care per 1000 Veterans associated with intervention [95% conf. int.]						
Two-way fixed-effects only[a]	5.8 [2.5, 9.1]	$p<0.001$	5.8 [1.5, 10.1]	$p=0.009$	6.3 [-0.5, 13.1]	$p=0.076$
Doubly-robust (simple)[b]	13.6 [7.3, 20]	n/a	11.8 [4.4, 19.2]	n/a	9.8 [2.9, 16.8]	n/a
Double-robust (dynamic)[c]	10.4 [5.5, 15]	n/a	9.5 [3.5, 16.5]	n/a	8.0 [2.5, 13.6]	n/a

a. OLS linear model with year-month and site fixed effects, and no covariates. Standard errors are clustered at the site level.
b. Mean effect aggregated across each group and time period, adjusting for covariates in eTable 2.
c. Summary of average treatment based on event-study/dynamic aggregation, adjusting for covariates in eTable 2.

© 2023 Cornell PY et al. *JAMA Network Open.*

eTable 5. Effects Grouped by Sites That Started the Staffing Program in the Same Month

Group Effects:					
Year-Month	Group	Estimate	Std. Error	[95% Simult. Conf. Band]	
2016-Nov	2	0.0256	0.0188	-0.0204	0.0717
2017-Jan	4	0.0043	0.0036	-0.0046	0.0132
2017-Feb	5	0.0044	0.0026	-0.002	0.0107
2017-Mar	6	0.0383	0.0178	-0.0052	0.0818
2017-Apr	7	0.1865	0.0493	0.0656	0.3075 *
2017-May	8	0.0153	0.0102	-0.0096	0.0402
2017-Jun	9	0.0226	0.0148	-0.0137	0.0588
2017-Oct	13	0.0127	0.0145	-0.023	0.0483
2017-Nov	14	0.0105	0.0148	-0.0257	0.0468
2017-Dec	15	0.0077	0.0055	-0.0057	0.0211
2018-Jan	16	0.072	0.0787	-0.1208	0.2649
2018-Feb	17	0.0243	0.0096	0.0006	0.0479 *
2018-Mar	18	0.037	0.0529	-0.0926	0.1666
Summary average treatment effect		0.0156	0.033	0.0092	0.022*
Signif. codes: `*' confidence band does not cover 0					
Control Group: Not Yet Treated, Anticipation Periods: 0					
Estimation Method: Doubly Robust					

© 2023 Cornell PY et al. *JAMA Network Open.*

eTable 6. Falsification Tests Using Inpatient Consults and Mortality as Outcomes

	Cohort	
All hospitalizations n=91,675	Any Hospitalization	
Inpatient palliative care consult ordered [a,b]	-1.9<br>[-53.3, 49.7]	p=0.94
30-day mortality[a]	-0.4<br>[-4.5, 3.6]	p=0.84

a. Adjusted for demographic and clinical characteristics listed in Table 1.
b. Consults identified from inpatient orders in the health record with associated stop codes of 351 or 353.

eFigure. Event Study and Evaluation of Parallel Trends

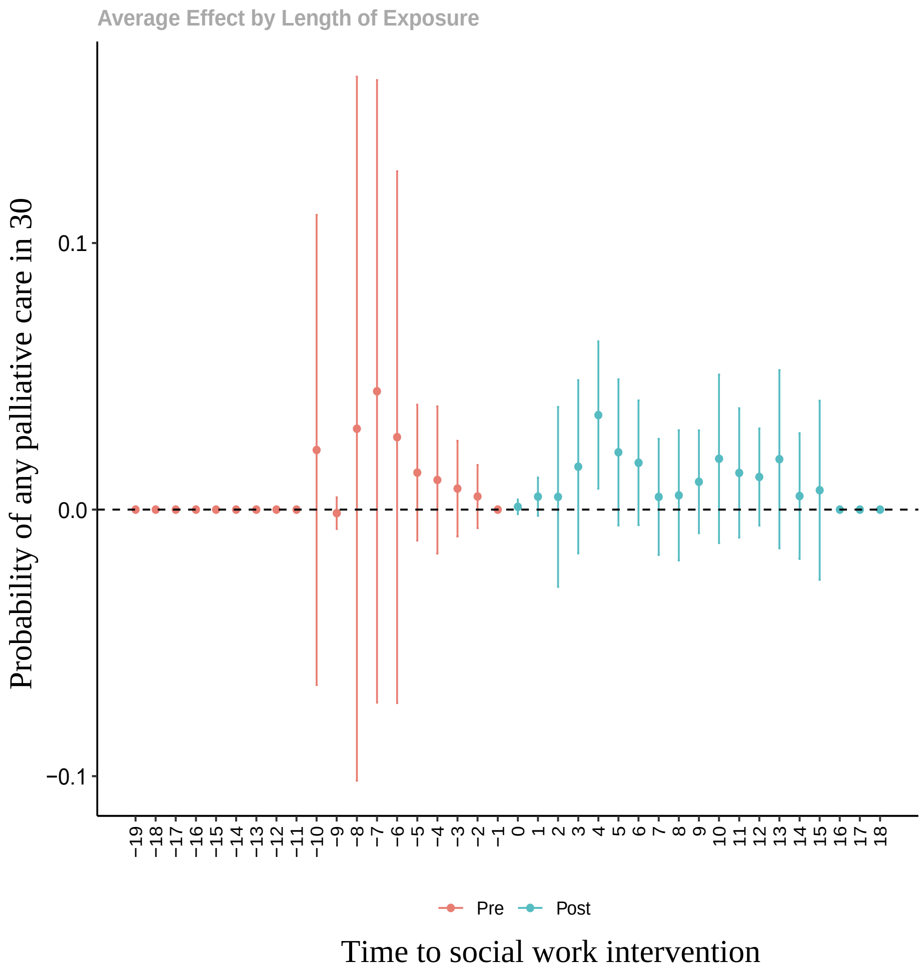

© 2023 Cornell PY et al. *JAMA Network Open.*
