# Supplementary material for: Social Work Staffing and Use of Palliative Care Among Recently Hospitalized Veterans
Source: JAMA Netw Open. 2023 Jan 4;6(1):e2249731. doi: 10.1001/jamanetworkopen.2022.49731 (PMC9856777; doi:10.1001/jamanetworkopen.2022.49731)
Supplement: Supplement 2. — Data Sharing Statement [file jamanetwopen-e2249731-s002.pdf]

Data Sharing Statement

Cornell. Social Work Staffing and Use of Palliative Care Among Recently Hospitalized Veterans. *JAMA Netw Open*. Published January 04, 2023.
doi:10.1001/jamanetworkopen.2022.49731

Data
Data available: No

Additional Information
Explanation for why data not available: Data are protected health information from the Veterans Affairs health system, and cannot be made available publicly.
